# Effectiveness and safety of acupuncture for treatment of tinnitus: A protocol for systematic review and meta-analysis

**DOI:** 10.1097/MD.0000000000022501

**Published:** 2020-10-02

**Authors:** Yepeng Yang, Qinwei Fu, Li Fu, Xuemei Wang, Juan Zhong, Qinxiu Zhang

**Affiliations:** a Hospital of Chengdu University of Traditional Chinese Medicine; b College of Clinical Medicine, Chengdu University of Traditional Chinese Medicine; c School of Medical and Life Sciences, Chengdu University of Traditional Chinese Medicine, Chengdu, Sichuan province, China.

**Keywords:** acupuncture, effectiveness, meta-analysis, safety, tinnitus

## Abstract

**Background:**

Tinnitus is a common disease in otolaryngology. In China, acupuncture has been used as a promising treatment for tinnitus. Yet, the specific effect and safety of acupuncture are still disputable. The ultimate goal of this paper is to formulate a protocol for systematic review and meta-analysis, which can be employed in assessing the benefits and safety of acupuncture on tinnitus.

**Methods:**

Seven databases should be retrieved from their establishment until June 2020, including PubMed, Cochrane Central Register of Controlled Trials, Excerpt Medical Database, Chinese Biomedical Literature Database, Chinese Science and Technology Periodical Database, China National Knowledge Infrastructure and Wan Fang Database. Randomized controlled trials of acupuncture treatment of tinnitus will be included. The experimental group is acupuncture or combined with additional treatment measures, and the control group is a placebo, sham acupuncture, Cognitive Behavioral Therapy, sound therapy, conventional medication, or same additional treatment. The clinical efficacy rate, Tinnitus Handicap Inventory, Tinnitus Questionnaire, visual analogue scale or other indicators are all concerned in the systematic evaluation of the program. Data collection, selection and extraction should be made separately by different researchers. The quality of the literature will be evaluated by the bias analysis table in the Cochrane Handbook, and Review Manager 5.3 software shall be applied to data analysis.

**Results:**

This protocol has made a concrete plan to evaluate whether acupuncture is effective and safe in curing tinnitus.

**Conclusion:**

This protocol is suitable for evaluating the effectiveness and safety of acupuncture in curing tinnitus, and is helpful for subsequent evaluation.

Open Science Framework Registration DOI: 10.17605/OSF.IO/85FCS.

## 1 Introduction

Tinnitus refers to the conscious ringing in the ear or brain in defect of external auditory stimulation.^[1]^ The incidence of tinnitus in adults is approximately10% to 15%,^[2]^ and it dramatically affects the work and life of some patients. Not surprisingly, tinnitus imparts an enormous economic and social burden.^[3]^ Because the aetiology and pathogenesis of tinnitus are not clear, evidence-based treatment options for tinnitus are still limited. There is currently no evidence that drugs specifically designed to treat tinnitus are valid, but studies suggest that these drugs have potentially dangerous side effects.^[1]^ Cognitive Behavioral Therapy (CBT) is efficient in reducing depression and improving quality of life.^[4]^ Unfortunately, it increases the direct cost of patients but does not affect lowering the subjective loudness of tinnitus. Moreover, CBT must be operated by professional practitioners, which may limit its applicability in general clinics. Therefore, people seek effective complementary and alternative therapies to treat tinnitus, and acupuncture may be one of the ways to resolve the matter.

In recent years, the treatment of tinnitus using acupuncture has shown its advantages and attracted increasing attention of researches. Acupuncture can effectively relieve the symptoms caused by tinnitus in patients, reduce the loudness and disability of tinnitus, and improve the hearing level.[^5^,^6^,^7^] The mechanisms of acupuncture treatment of tinnitus may include several aspects:

1. By directly stimulating local acupoints, acupuncture can strengthen local blood circulation, improve metabolism and promote unobstructed qi and blood in the ears, then the tinnitus is relieved.^[8]^
2. Acupuncture can reduce abnormal electrical activities in the cochlea, leading to active reorganization of the auditory cortex to better perceive tinnitus signals. Acupuncture may cause functional restructuring of the superior frontal gyrus so that the bad mood of tinnitus patients can be improved.^[9]^
3. Acupuncture has been proven to adjust the central neurotransmitter γ-aminobutyric acid, 5-hydroxytryptamine and the state of their receptors, thereby inhibiting or exciting specific components of the auditory system.^[10]^

A systematic review that covered 11 randomized controlled trials(RCTs) was published in 2016, but no convincing evidence that acupuncture is beneficial in the treatment of tinnitus was reported in that study.^[11]^ Moreover, there is still no recommendation concerning the treatment of tinnitus with acupuncture in the latest European multidisciplinary tinnitus guidelines. Aiming to encourage researchers to concern the acupuncture treatment in tinnitus, a high-quality systematic evaluation program that can be used to assess the safety and availability of acupuncture in curing tinnitus is formulated in the present study.

## 2 Methods

This study protocol has been enrolled in Open Science Framework (OSF), with the registration DOI 10.17605/OSF.IO/85FCS (https://osf.io/85fcs). We follow the Preferred Reporting Items for Systematic Reviews and Meta-analysis Protocol (PRISMA-P) ^[12]^ to accomplish the systematic review protocol. This study is for the secondary collection and analysis of original RCTs data, so ethical approval or patient informed consent is not demanded.

### 2.1 Inclusion and exclusion criteria

#### 2.1.1 Research types

RCTs for tinnitus treatment with acupuncture should be included. Language is limited to English and Chinese. Duplicate publications, no controls, literature reviews, case reports, case series, animal experiments and other irrelevant articles should be eliminated.

#### 2.1.2 Participants

Patients with tinnitus who meet diagnostic criteria regardless of sex, age, race, or severity.

#### 2.1.3 Interventions

Patients in the experimental group are treated by acupuncture, including body acupuncture, electroacupuncture, scalp acupuncture, and ear acupuncture. There are no restrictions on acupuncture materials, acupuncture points, stimulation methods, retention time, and treatment duration. Patients in the control group are given 1 or several of the following treatments, including a placebo, sham acupuncture, CBT, sound therapy or conventional medication.

Suppose a study makes a comparison between the experimental group that adopts acupuncture in combination with another intervention and the control group that chooses only another intervention the same as the experimental group; in that case, the study should also be included. Studies that only compare the therapeutic effects of different forms of acupuncture (for instance, comparing body acupuncture and electroacupuncture) will be excluded.

#### 2.1.4 Outcome indicators

##### 2.1.4.1 Main outcome indicators

1. Self-report tinnitus questionnaire: including Tinnitus Handicap Inventory (THI), Tinnitus Questionnaire (TQ), Tinnitus Reaction Questionnaire (TRQ), Tinnitus Handicap Questionnaire (THQ), Tinnitus Function Index (TFI) 5 kinds. Besides, the tinnitus evaluation questionnaire ^[13]^ developed by the Chinese scholar Liu Peng will be employed to assess the efficacy of tinnitus. In each study, only 1 of them or a combination of 2 scales may be employed.
2. Visual analogue scale (VAS)
3. Clinical efficacy rate

##### 2.1.4.2 Secondary outcome indicators

The secondary indicators should include Hamilton Anxiety Scale (HAS), Beck Depression Inventory (BDI), pure tone audiometry (PTA), otoacoustic emissions (OAEs) and adverse reaction (including fainting on acupuncture, bent needle, stuck needle, broken needle, infection and hematoma).

### 2.2 Search strategy

Seven electronic databases shall be retrieved from their establishment until June 2020: PubMed, Cochrane Central Register of Controlled Trials (CENTRAL), Excerpt Medical Database (Embase), Chinese Biomedical Literature Database (CBM), Chinese Science and Technology Periodical Database (VIP), China National Knowledge Infrastructure (CNKI) and Wan Fang Database. The database retrieval strategies are displayed in Table 1. We will try to identify other studies by tracing the references of related trials, manually searching associated journals, and collecting grey literature. If necessary, we will communicate with the authors for more detailed information.

**Table 1:**
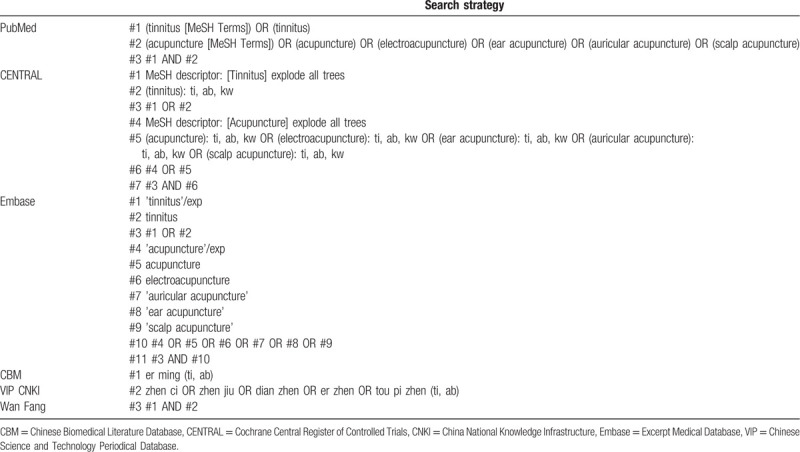
Search strategy for each database.

### 2.3 The collection and analysis of the data

#### 2.3.1 Data selection

**Figure 1:**
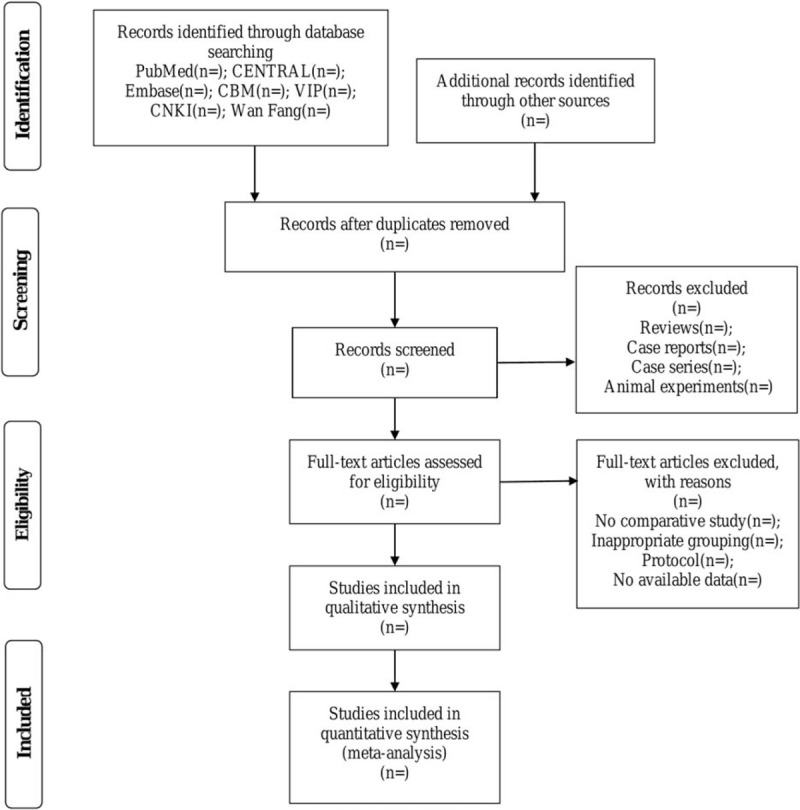
Flowchart of study selection.

The Endnote X8 software will be adopted to remove duplicate documents. We have 5 researchers to review correlative literature. Two reviewers (Yang YP and Fu QW) will first conduct a preliminary screening based on the inclusion and exclusion criteria and exclude documents that do not meet the requirements by reading the title and summary of the study. Then 2 other reviewers (Fu L and Zhong J) will read the full text of the paper that passed the preliminary screening to determine whether they should be included or not. For uncertain studies, the assessment will be made by the third reviewer (Zhang QX). The literature will be filtered in the light of the PRISMA flow diagram (Fig. 1).

#### 2.3.2 Extraction and management of the data

Data including baseline data (such as age, gender, course of the disease), intervention data (such as treatment methods, frequency, course of treatment), outcome data and quality data (such as the generation of random sequences, allocation concealment and blinding) will be extracted by 2 reviewers (Yang YP and Fu QW) independently. We will resolve disagreements among literature by discussing with Zhang QX for further determination. For incomplete data, we shall contact the writer for specific information.

#### 2.3.3 Risk of bias assessment

The potential risk of bias in each research shall be evaluated severally by 2 evaluators (Yang YP and Wang XM) following the quality standards in the Cochrane Handbook for Systematic Reviews of Interventions (version 5.1.0).^[14]^ Any dissension should be solved in consultation with a third reviewer (Zhang QX). Six bias items are as follows:

1. Generation of random sequences;
2. An adequate allocation and conceal the course;
3. Blinding of the subject, observer, and statistical analysis;
4. Incomplete result data;
5. Selective reporting results and other sources of bias.

#### 2.3.4 Statistical analysis

Review Manager 5.3 shall be used to conduct data analysis. The relative risk (RR) and mean differences [MD, with 95% confidence interval (95%CI)] will be adopted as statistical indicators in the analysis of dichotomous data and continuous data, respectively. If studies are measured their outcomes in different ways, the standardized mean difference (SMD) will be applied.

Statistical heterogeneity should be evaluated by Chi-Squared tests and *I* ^2^ statistic. In the heterogeneity test, *P* > .1 or *I* ^2^ < 50% means no apparent heterogeneity, and the fixed effects model will be elected. *P* < .1 or *I* ^2^ > 50% indicates significant heterogeneity, a random-effect model or subgroup analysis can be used. If heterogeneity may come from factors such as country and region, race, age, gender, treatment duration, we will use subgroup analysis for analysis. Alternatively, we will remove low-quality studies and use sensitivity analysis to investigate which study has the most significant impact on heterogeneity. If quantitative synthesis is not possible, we will make a qualitative description.

If more than ten trials report the same results, the funnel plots will be employed to assess publication bias. After the data analysis is completed, the grades of Recommendations Assessment, Development and Evaluation profiler (GRADEpro) software shall be applied to estimate the quality of the evidence.

## 3 Discussion

Tinnitus is known as one of the 3 major problems in otology (tinnitus, deafness, and dizziness), which may damage patients mood, cognition, concentration, and even cause serious health problems such as depression, anxiety and insomnia.^[15]^ Due to the accelerated pace of life, increased life pressure and ageing, people are paying more attention to the treatment of tinnitus.

Traditional Chinese medicine holds that the ear is close to the sanjiao meridian of hand-shaoyang, the xiaochang meridian of hand-taiyang and the gallbladder meridian of foot-shaoyang. Acupuncture can better improve tinnitus by stimulating the meridian points around the ear. As far back as Huangdi neijing, there have been records of acupuncture therapy of tinnitus. In clinical practice, a large number of doctors choose acupuncture as a therapeutic method for tinnitus. [^16^,^17^,^18^] At present, acupuncture treatment of tinnitus has been reviewed systematically, but convincing evidence has not been provided, which limits the application of acupuncture. Therefore, it is essential to supplement the latest randomized controlled trial studies in further work to estimate the availability and safety of acupuncture in curing tinnitus correctly. We harbour the idea that the protocol reported in this study can provide clinicians and researchers with some help for exploring acupuncture treatment of tinnitus.

## Acknowledgments

We thank Dr Xinsen Wei, Capital Normal University, for guiding the revision of the paper.

## Author contributions

**Conceptualization:** Yepeng Yang, Qinxiu Zhang.

**Investigation:** Yepeng Yang, Qinwei Fu, Li Fu, Xuemei Wang, Juan Zhong.

**Methodology:** Yepeng Yang, Qinwei Fu, Li Fu, Xuemei Wang.

**Project administration:** Yepeng Yang, Qinxiu Zhang.

**Supervision:** Yepeng Yang, Juan Zhong.

**Writing – original draft:** Yepeng Yang, Qinwei Fu.

**Writing – review & editing:** Yepeng Yang, Qinxiu Zhang.
